# The Influence of Ag on the Microstructure and Properties of Cu-Ni-Si Alloys

**DOI:** 10.3390/ma13153416

**Published:** 2020-08-03

**Authors:** Beata Krupińska, Wojciech Borek, Mariusz Krupiński, Tatiana Karkoszka

**Affiliations:** Department of Engineering Materials and Biomaterials, Faculty of Mechanical Engineering, Silesian University of Technology, Konarskiego St. 18a, 44-100 Gliwice, Poland; wojciech.borek@polsl.pl (W.B.); mariusz.krupinski@polsl.pl (M.K.); tatiana.karkoszka@polsl.pl (T.K.)

**Keywords:** copper alloys, heat treatment, plastic deformation, strengthening, conductivity

## Abstract

The influence of the mass concentration of Ag on properties of Cu-Ni alloys is investigated. The effect of silver addition on the structure and properties of Cu-2Ni-1Si alloys is determined. The scientific aim of this research is to determine how the addition of silver affects the mechanisms of strengthening silver-modified supersaturated, deformed, and aged Cu-2Ni-1Si alloys. The applied thermo-derivative analysis has allowed us to determine a range of the temperature values for the beginning and the end of crystallization, the phases and eutectics, and the effects of the modification on the solid fraction of the solidified alloy. In addition to the crystallization kinetics, the microstructure morphology, mechanical properties under real operating conditions, and the electrical conductivity have also been investigated. Moreover, the conducted research includes the impact of heat treatment and plastic deformation on the alloy structure and considers the type, share, and distribution of the intermetallic phases and structural stresses caused by coherent phases, as well as the effect of dislocations in the reinforcing phases during aging. Electron microscopy (SEM), micro-area analysis (EDS), optical microscopy, hardness measurements, and conductivity of the tested alloys are utilized to comment on these properties.

## 1. Introduction

The drive for industrial advancement and new technologies forces the development of materials engineering and the continuous improvement of existing materials, as well as the development of completely new classes of high-quality materials [1,2,3,4]. Operational durability and reliability are a priority and drive the search for materials with more favorable and precise sets of features, such as high and stable mechanical, electrical, and/or thermal properties during operation. With the development of both new technologies and new possibilities for conducting scientific research, the creation of such materials is becoming more facile. Copper alloys are examples of such materials [5,6,7].

Thermal-derivative analyses of solidifying alloys aim to determine the temperature of phase transitions during the cooling processes. It allows one to define the temperature of both the beginning, T_L_, and the end of crystallization, T_sol_ [8,9,10,11]. Thermal-derivative analysis (TDA), with the use of the UMSA MT5 platform, measures the temperature change during solidification of the molten metal. The total latent heat and fraction solid can then be calculated by differentiating the cooling curve. A critical requirement for the cooling curve analysis is the determination of “the baseline”.

Some of the properties, such as the high and stable mechanical, electrical, and thermal properties during operation, are observed in copper alloys hardened by precipitation, such as Cu-Fe, Cu-Cr, Cu-Co, Cu-Ni-Si, and Cu-Ni-Si-Cr [1,2,3,4]. The Cu-2Ni-1Si alloy’s mechanical properties can be shaped by heat and plastic treatment, and these properties mean it is subjected to significant research [12,13,14,15]. In this alloy, Cr, Cr and Mg, or Cr and Ti are often introduced to obtain Cu-Ni-Si-Cr, Cu-Ni-Si-Cr-Mg, or Cu-Ni-Si-Cr-Ti alloys, doping of these elements causes phase transformations and morphology changes and, consequently, results in more favorable mechanical properties and can cause changes in the conductivity in some cases [1,2,3,4].

In the investigated Cu-2Ni-1Si alloys [1,6], supersaturated at a temperature of 950 °C, a broad scope of changes in mechanical properties and, in some, changes in electrical conductivity are shown. It is closely associated with the structure formed due to heat treatment and cold plastic deformation. In the research [6], a significant role of nanometric, coherent precipitates created as the result of supersaturating and aging of Cu-2Ni-1Si alloy has been indicated. The precipitates (Ni_2_Si) grow as the aging time progresses and the distance between them also increases. Precipitation of Ni_2_Si particles has an intensive character at the time of aging, and it goes homogeneously in the matrix over a temperature range of 267–381 °C [6]. The changes in the morphology of the precipitates in the matrix accompany changes in mechanical properties and changes in the electrical conductivity [6].

Previous work [7] has shown that the isothermal Cu-rich phase system, at temperatures ranging from 300 °C to 600 °C, there are three two-phase ranges and two three-phase ranges. These three two-phase ranges are: FCC-Al (Cu-rich) + γ-Ni_5_Si_2_, FCC-Al (Cu-rich) + δ-Ni_2_Si, and FCC-Al (Cu-rich) + ε-Ni_3_Si_2_. The two three-phase ranges are: FCC-Al (Cu-rich) + γ-Ni_5_Si_2_ + δ-Ni_2_Si, and FCC-Al (Cu-rich) + δ-Ni_2_Si + ε-Ni_3_Si_2_. For this reason, in the Cu-rich alloys, the Cu phases of γ-Ni_5_Si_2_, δ-Ni_2_Si or ε-Ni_3_Si_2_ can be precipitated; the proportion of each precipitated phase is dependent on both the alloy composition and the aging temperature. Transmission electron microscopy analysis of the alloy Cu-3.2Ni-0.75Si suggests that they contain a δ-Ni_2_Si phase with only a few γ-Ni_5_Si_2_ phase particles—this is consistent with the thermodynamic calculation of equilibrium.

Research has shown [16] that the properties of the Cu-Ni-Si in Cu-Ni-Si-Ag alloys after plastic deformation and heat treatment can be obtained. The obtained microstructure is dependent on the temperature and strain rate, whilst the Ag addition causes both a structural refinement and recrystallization acceleration. It has been shown that the increase in aging temperature increases the microhardness and conductivity [16,17,18,19,20].

The development of research methods concerning the microstructure of alloys, as well as the mechanical properties describing the fracture mechanics regarding testing and characterization of materials, also in the nanoscale, allows for modern material characterization [1,2,7,13,14,21].

Cu-Ni-Si alloys are strengthened with the introduction of δ-Ni_2_Si and ε-Ni_3_Si_2_ phases. We have previously shown that 1 mass% addition of Ag influences the derivation curve of the solidifying alloy, supersaturating and aging of the cold-worked Cu alloys. The impact of silver addition on the precipitation strengthening leads to an improvement in the mechanical properties and conductivity of the alloy.

An understanding of the phenomena occurring in the solidified Cu alloys is important for forming the microstructure and the design of alloys with desirable functional properties. These determined properties also affect the process parameters for heat and plastic treatment, such as temperature and time. The addition of 0.8 mass% Ag causes a precipitate strengthening of the alloy and, therefore, the hardness increases by 22% with a simultaneous conductivity increase.

## 2. Materials and Methods

**Figure 1:**
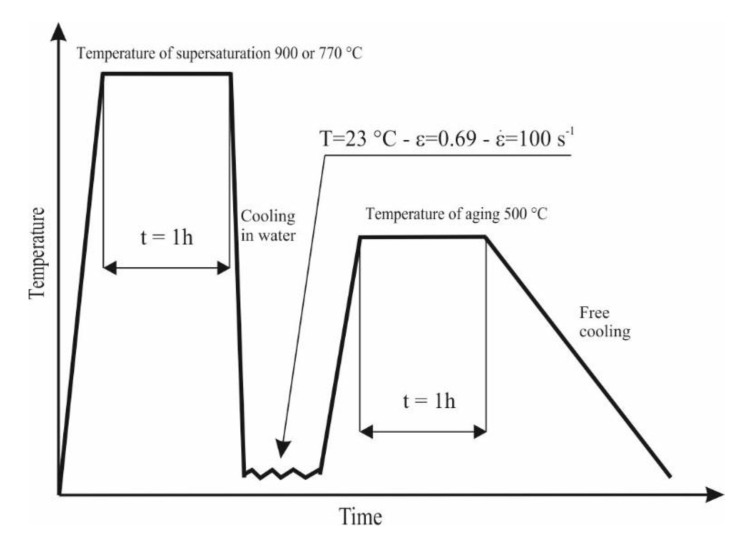
Diagram of the heat treatment and cold plastic deformation of investigated alloys Cu-2Ni-1Si (supersaturating at temperature 900 °C) and Cu-2Ni-1Si-0.8Ag (supersaturating at temperature 770 °C).

In this paper, we focus on two alloys Cu-2Ni-1Si and Cu-2Ni-1Si-0.8Ag, whose chemical compositions are given in Table 1. The 22 different samples were tested and results were analyzed. The investigated alloys were subjected to heat treatment and subsequent cold plastic deformation (Figure 1).

The investigated heat treatment process was based on the supersaturation of Cu-2Ni-1Si-0.8Ag alloys at a temperature of 770 °C and for the Cu-2Ni-1Si alloys a temperature of 900 °C, which were followed by aging at a temperature of 500 °C. A DSI (Dynamic System Inc., Poestenkill, NY, USA) thermomechanical simulator Gleeble 3800 (Dynamic System Inc., USA) hosted by the Department of Engineering Materials and Biomaterials of the Silesian University of Technology was used. Both heat treatment and cold plastic deformation were completed using the Gleeble 3800 (Dynamic System Inc., Poestenkill, NY, USA). The Gleeble 3800 simulator is equipped with a direct resistance heating system, which accurately maintains the desired temperature with an accuracy of 1 °C. The application of the Gleeble simulator allows us to perform the heat treatment at a precise, predetermined temperature and thereby minimize errors during heating, annealing, and direct cooling of the pressurized water stream or compressed air during the process. To protect the samples from any unintended connection with the tungsten carbide anvil and also to improve the interaction between the contact surfaces (which is very important in terms of resistive heating realized in the simulator and involves a current flow by the tested sample), as well as to reduce the friction between the sample’s surface and the tungsten carbide anvils, a set of graphite and tantalum foils 0.1 mm thick were used. The contact surfaces of the sample and the anvils were also covered with a nickel-based lubricating grease.

The first stage of the heat treatment involves supersaturating the tested alloys, the cylindrical samples with dimensions of Ø 12 mm × 12 mm were resistively heated in an argon atmosphere at the rate of 3 °C/s to a set temperature of 770 °C for Cu-2Ni-1Si-0.8Ag alloys and at a temperature of 900 °C for the Cu-2Ni-1Si alloys. The alloys were annealed at a suitable temperature for 1 h, and then cooled with a stream of water at 50 PSI (350 kPa). Water was sprayed from 4 nozzles directly onto the sample for 20 s, which allowed the sample to cool to room temperature (about 20–30 °C). After the supersaturated samples were subjected to an intense cold plastic deformation process with a very high deformation rate of 100 s^−1^. The deformation value was 50% (sample height before deformation—12 mm, sample height after deformation—6 mm, strain = 0.69, determined as ln(final length/initial length). Cold plastic deformation showed large deformation of the alloy’s structure and was observed at a room temperature (~23 °C) under a protective atmosphere of argon. For the next stage, the samples were subjected to an aging process at an initial temperature of 500 °C (the heating rate of the samples was set to 3 °C/s), which was completed for 1 h under a protective argon atmosphere. For the heat treatment or cold plastic deformation experiments, before the introduction of the protective gas into the working chamber of the Gleeble 3800 simulator, first, a vacuum of 0.1 mBar was created in the chamber, and then the argon protective gas with a partial pressure of approximately 200 mBar was introduced.

As part of the performed tests, we can distinguish, among others:

- Samples prepared for observation of the microstructure were ground and polished mechanically, then electropolished and etched in an electrochemical reagent or using one of the following reagents: iron chloride, hydrochloric acid, and ethyl alcohol.
- The thermo-derivative analysis was completed with the application of a UMSA (Method and Apparatus for Universal Metallurgical Simulation and Analysis-Patent Serial No. PCT/CA02/01903, Silesian University of Technology, Gliwice, Poland) device equipped with a computer-controlled cooling system, which allows flexible setting of the cooling rate applied for Cu-2Ni-1Si and Cu-2Ni-1Si-0.8Ag alloys. The samples for thermo-derivative analysis were prepared with a diameter of Ø 8 mm and a height of 10 mm. Holes for thermocouples (type K) were made in the samples, where the thermal node occurs for this type and arrangement of the sample geometry.
- Microstructure and chemical composition were investigated with EDS microanalysis using a scanning electron microscope Zeiss Supra 25 and an MEF4A (SEM, Thornwood, NY, USA) optical microscope supplied by Leica together with their image analysis software.
- Hardness tests were performed with a hardness tester Vickers Future-Tech (FM-ARS 9000, FM-ARS9000, Future-Tech, Tokyo, Japan) with a load of 1000 gf for 10 s.
- Electrical conductivity was measured using a Sigmatest Foerster device (FOERSTER, Pittsburgh, PA, USA).

## 3. Results and Discussion

**Figure 2:**
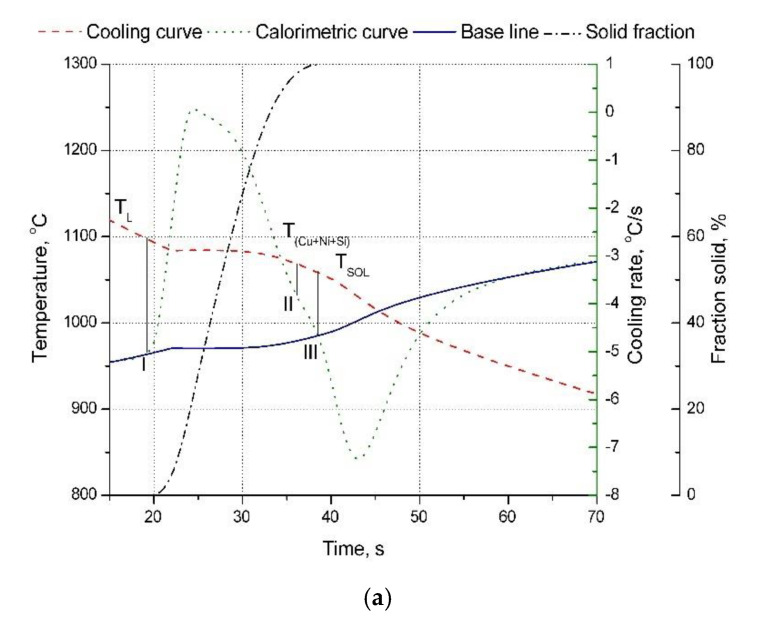

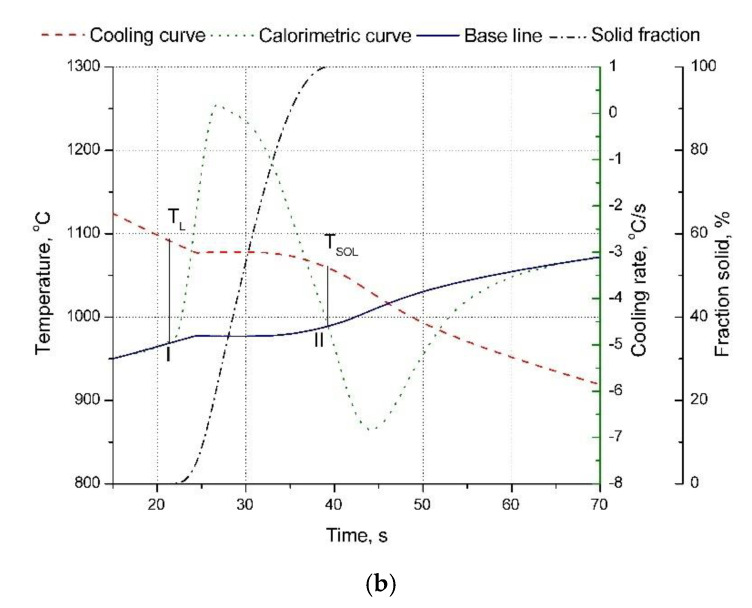
The cooling and derivative curves for the Cu-2Ni-1Si alloys. The solid fraction during the crystallization of the particular compounds of the Cu-2Ni-1Si alloy (**a**). The cooling and derivative curves for Cu-2Ni-1Si-0.8Ag alloys. The solid fraction during the crystallization of the particular compounds of the Cu-2Ni-1Si-0.8Ag alloy (**b**).

**Figure 3:**
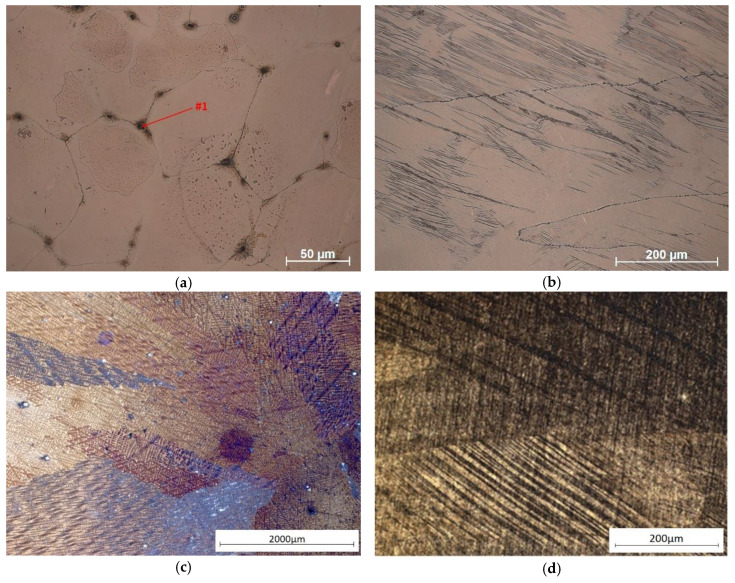
The microstructure of Cu-2Ni-1Si alloy casting state 25×, #1—Ni_2_Si phases (**a**)**,** the state after the heat and cold plastic treatment 200× (**b**)**,** the microstructure of Cu-2Ni-1Si-0.8Ag alloy casting state 25× (**c**), and the state after the heat and cold plastic treatment 200× (**d**).

**Figure 4:**
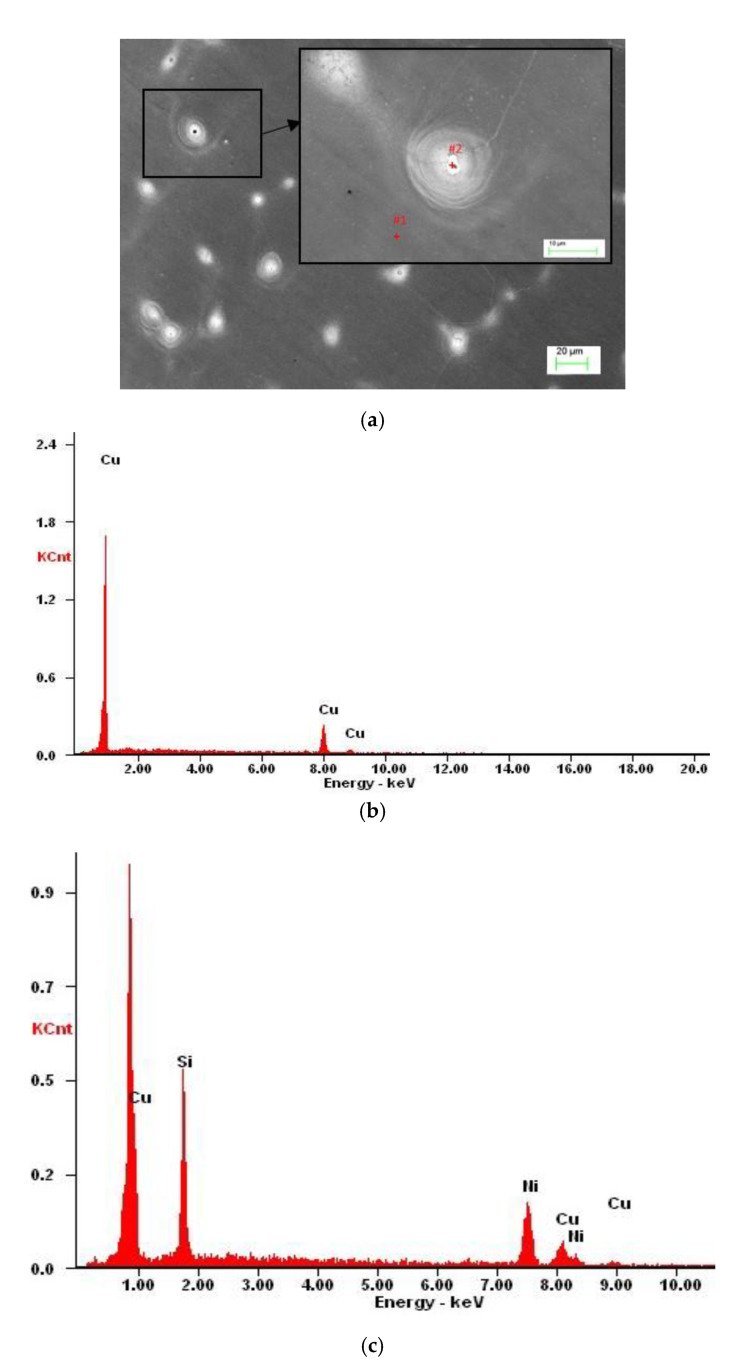
The microstructure of the Cu-2Ni-1Si alloy (casting state) (**a**), the EDS from the point #1 (Table 2) (**b**), and the EDS from the point #2, phases Ni_2_Si (Table 2) (**c**).

**Figure 5:**
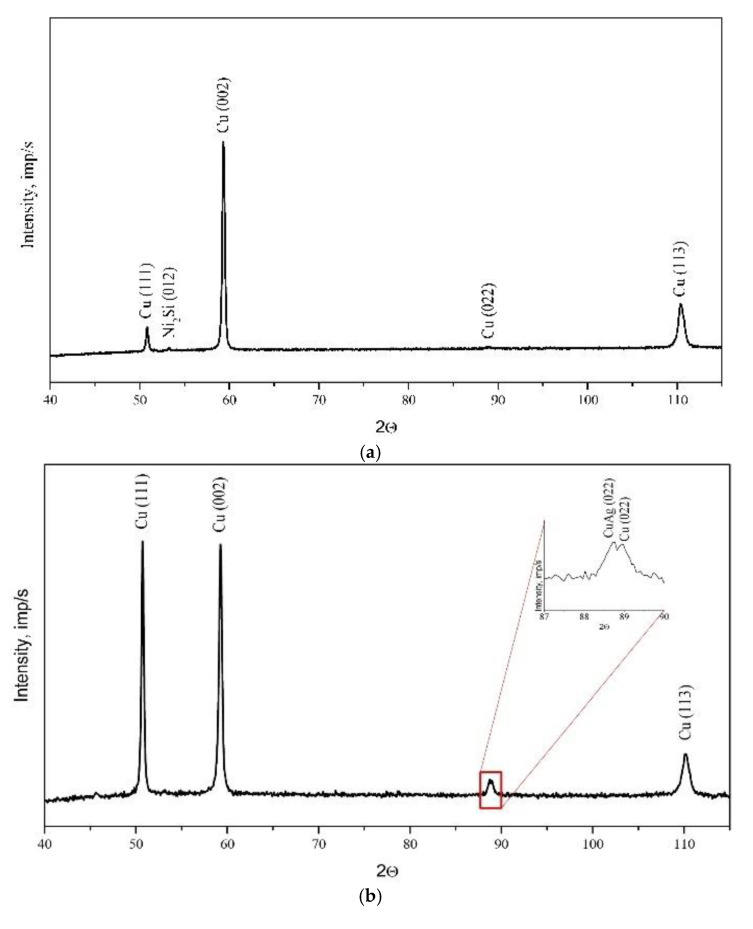
X-ray analysis of the substrate material before (**a**) and after Ag alloying (**b**).

From the analysis of the derivation curve (Figure 2a), it can be observed that at the time of solidification of Cu-2Ni-1Si at a temperature of 1080 °C at the point I (T_L_ = 1098 °C) the solidification of α phase occurs. At point II, it is followed by solidification of the Cu + Ni + Si eutectic. After analysis of the results using light microscopy, the presence of small, elongated Ni_2_Si intermetallic phases on the α phase boundaries can probably be observed (Figure 3a and Figure 4, Table 2), as well as confirmed by X-ray examinations (Figure 5) [1,2,6,7,22].

The temperature at the end of alloy solidification, based on thermal-derivative analysis, is equal to T_SOL_ = 1057 °C for the Cu-2Ni-1Si alloy (point III, Table 3, Figure 2a). For the alloy with the addition of silver at a concentration of 0.8 mass% (Table 3, Figure 2b), an increase in the solidification temperature of α phase to T_L_ = 1091 °C—in other words, an increase of 11 °C—is identified. The temperature at the end of solidification is T_SOL_ = 1060 °C, given as point II on Figure 2b. Based on the performed thermal-derivative analysis, the beginning and the end of solidification of phases and eutectics and any adequate temperatures cannot be discretely established.

The calculated values of the heat capacity in the liquid state Cpl and values of heat capacity in the solid-state Cps, respectively, for alloys Cu-2Ni-1Si and Cu-2Ni-1Si-0.8Ag, taking into account the change in cooling rate, have determined changes of crystallization heat of the structural elements, which has been shown in Table 4 and Table 5.

The changes in the chemical composition and phase composition that occurred as a result of the modification of copper with silver were examined by X-ray structural analysis, and the results are presented in Figure 5.

**Figure 6:**
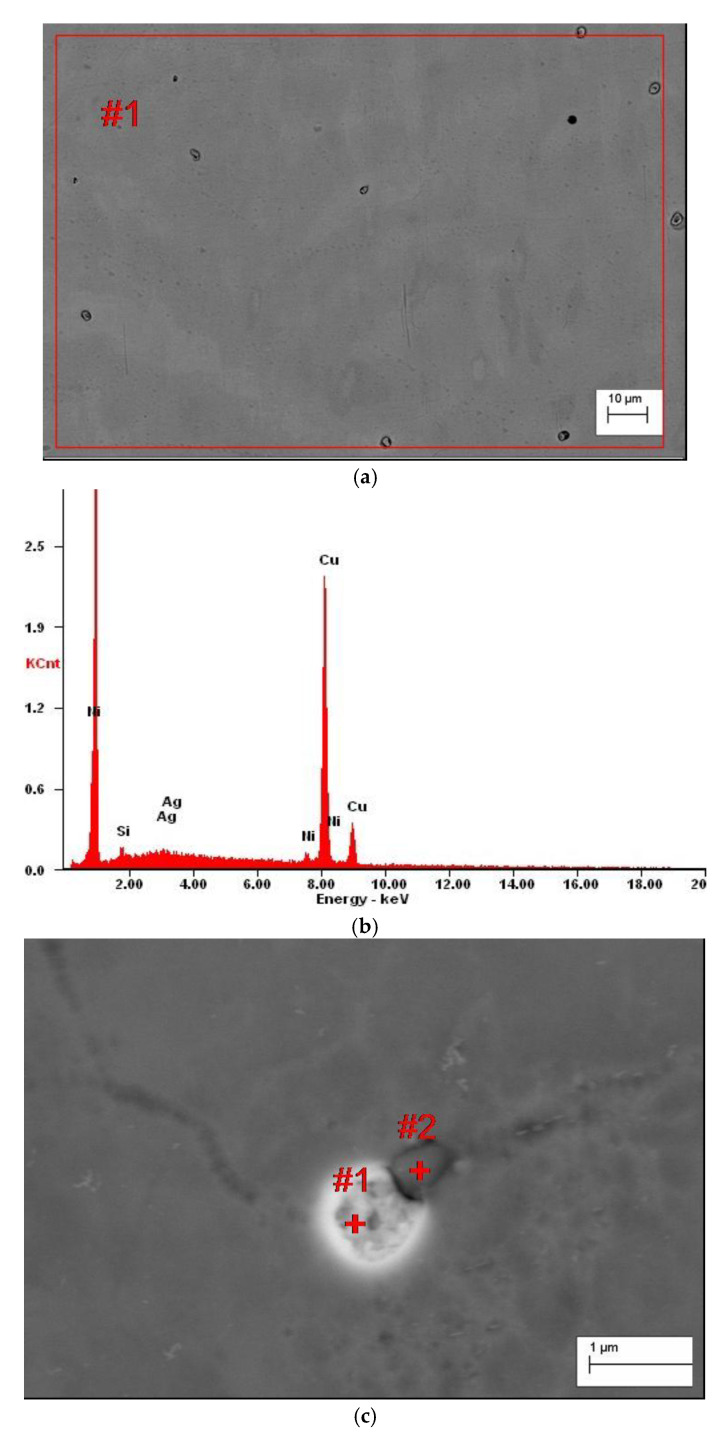

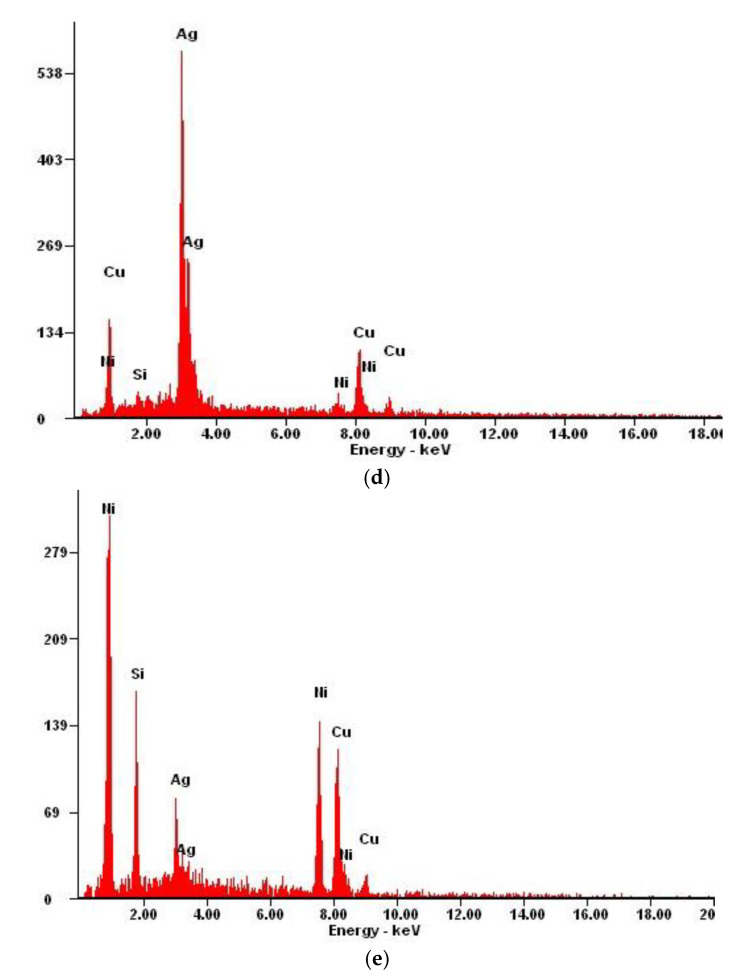
The microstructure of the Cu-2Ni-1Si-0.8Ag alloy (casting state) (**a**), the EDS from the micro-area #1(Figure 6a) (Table 6) (**b**), the microstructure of Cu-2Ni-1Si-0.8Ag alloy (casting state) (**c**), analysis of EDS from the point #1 (Figure 6c) (Table 6) (**d**), and the EDS from the point #2, phases Ni_2_Si (Figure 6c) (Table 6) (**e**).

**Figure 7:**
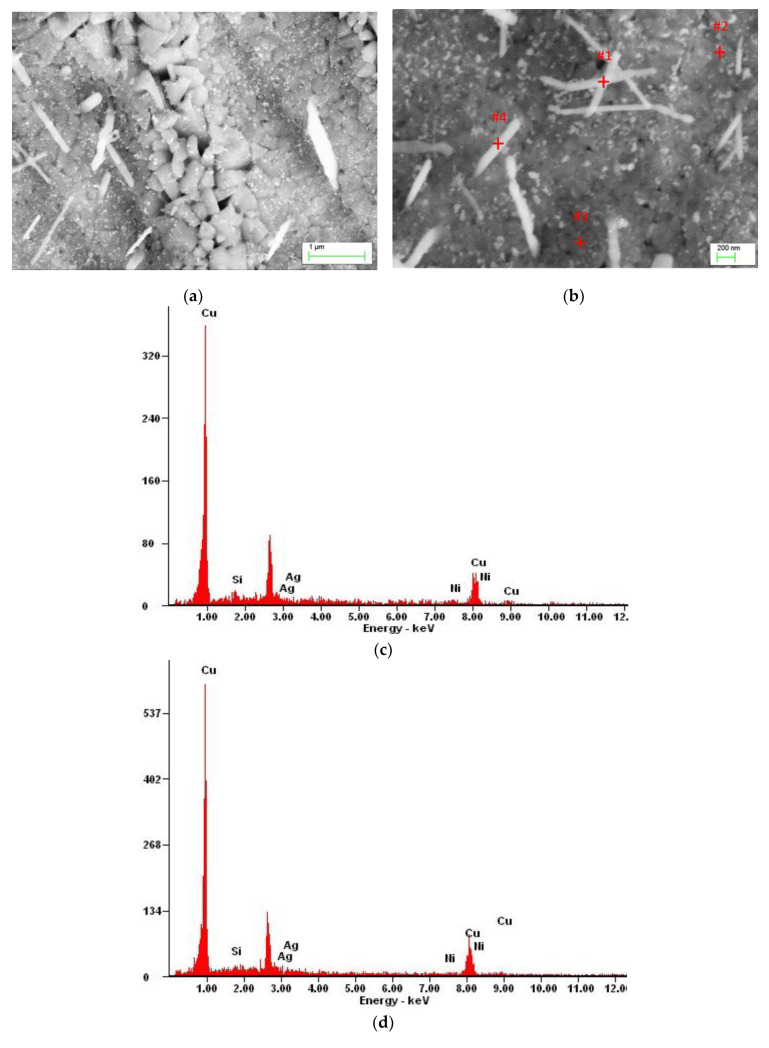

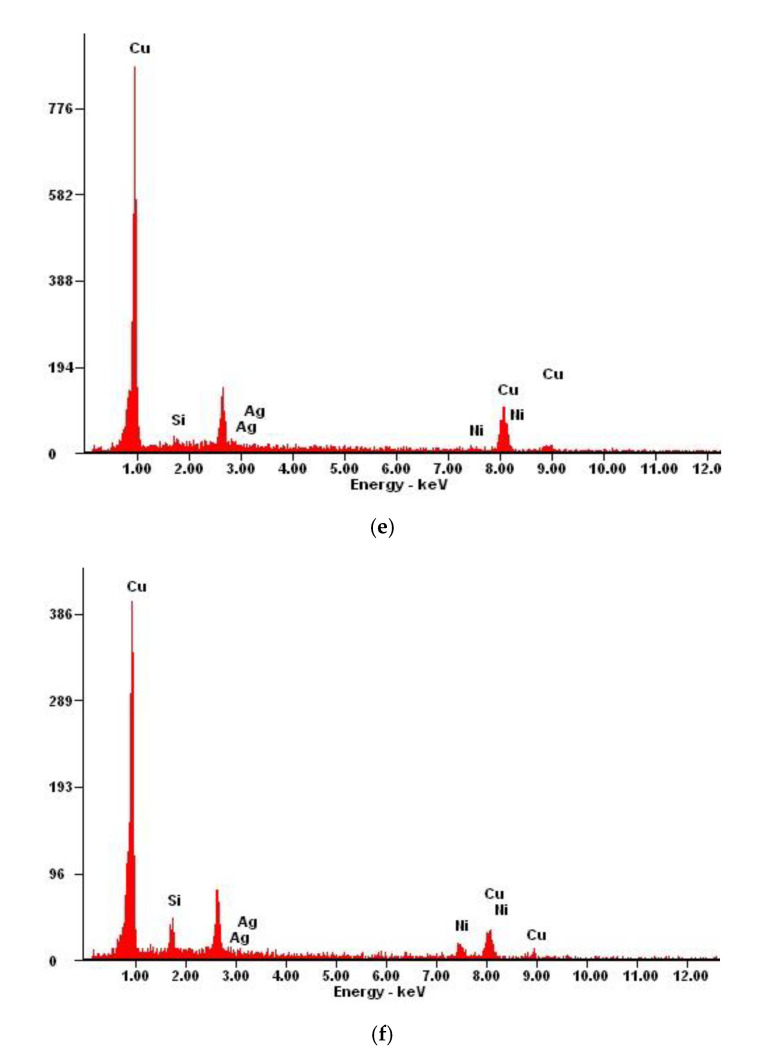
Structure of Cu-2Ni-1Si-0.8Ag alloy: supersaturation 770 °C; time 1h, cooling in water; cold plastic deformation 50%; aging 500 °C 1 h (**a**), structure of Cu-Ni-Si-Ag alloy: supersaturation 770 °C; time 1h, cooling in water (Ar); cold plastic deformation 50%; aging 500 °C 1 h (**b**), analysis of EDS from the point 1#, phases Ni_2_Si (Figure 7b) (Table 7) (**c**), analysis of EDS from the point 2# (Figure 7b) (Table 7) (**d**), analysis of EDS from the point 3# (Figure 7b) (Table 7) (**e**), and analysis of EDS from the point 4#, phases Ni_2_Si (Figure 7b) (Table 7) (**f**).

This implies that in Cu-2Ni-1Si-0.8Ag alloy α matrix starts to solidify in the T_L_ temperature, while the overcapacity of silver solidifies the intermetallic Ni_2_Si phase. The size of the eutectic is approximately 0.5 µm. The residual silver is dissolved in the matrix of the α phase, which has been observed by EDS (Figure 6 and Figure 7, Table 6 and Table 7).

**Figure 8:**
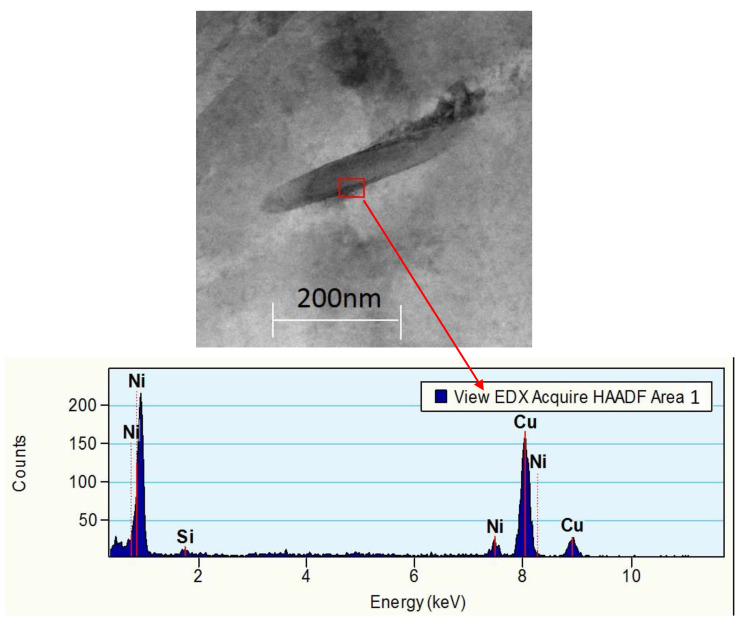
Structure of the Cu–2Ni–1Si alloy modified with Ag after heat treatment and cold plastic deformation, analysis of 1 zone made with energy-dispersive X-ray spectroscopy.

Tests using X-ray scanning transmission electron microscopy (STEM) with the application of chemical composition analysis with an EDS detector confirm the occurrence of phase Ni_2_Si in the Cu-2Ni-1Si-0.8Ag alloy (Figure 8).

For Cu-2Ni-1Si alloys, we applied supersaturation from a temperature of 900 °C. In the case of the Cu-2Ni-1Si-0.8Ag alloys, a visible flow of the material during the annealing process appeared at 900 °C. Due to the application of the direct resistance heating and effect of current flow across the sample in the Gleeble simulator, problems concerning changes in temperature resulting from a loose contact between the sample and the anvil have been noted. The pressure of anvils applied to the samples was slightly increased resulting in a free flow of the samples and sample melting, especially at the sample–anvil contact. An increase in the contact force resulted in an increase in the stress of the material and melting Cu + Ag eutectics caused deformation of the analyzed samples outside the mechanisms of slip and twinning (flow along grain boundaries). Based on these analytical results and the phase equilibrium diagram, the supersaturating temperature of the Cu-2Ni-1Si-0.8Ag alloy was lowered to 770 °C.

An excess of silver locally crystallized on the Ni_2_Si phase (Figure 6) due to the treatment causing silver to dissolve in α groundmass after aging participates. The Ni_2_Si phase, in turn, changed the morphology and took the form of stamens (Figure 7 and Figure 8).

**Figure 9:**
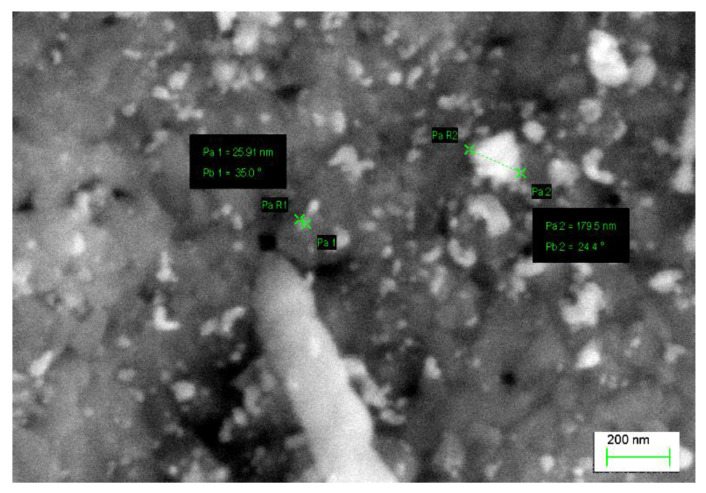
Structure of Cu-2Ni-1Si-0.8Ag alloy: supersaturation 770 °C; time 1 h, cooling in water; cold plastic deformation 50%; aging 500 °C 1 h.

Supersaturation of the analyzed Cu-2Ni-1Si-0.8Ag alloy was followed by even dissolution of silver in the α groundmass. The application of intensive plastic deformation with a draught coefficient value of 50% and a very high rate of shear ranging, 100 s^−1^, caused significant changes in the crystalline structure of the alloys. It is connected with massive increases in different crystal defects, especially dislocations. During cold plastic deformation with a draught coefficient and rate of shear of this size, the density of the point defects, which increase silver diffusion, also grew. During aging at a temperature of 500 °C, the precipitation of small (20 to 180 nm) strengthening Ag phases was observed (Figure 6c, Figure 7 and Figure 9). These phases can also be seen in Figure 7. The Ni_2_Si phases have the form of long phases with varying space orientation—this is observed in the EDS analysis (Figure 7).

The process of aging for 1 h at a temperature of 500 °C did not allow restoration from static recovery of the proper crystalline structure of the Cu-2Ni-1Si and Cu-2Ni-1Si-0.8Ag alloys after cold plastic deformation.

The aging temperature was utilized to determine the processes involved in elucidating the recrystallization temperature. The recrystallization temperature is an abstract concept and cannot be univocally defined, as it is dependent on many factors, such as the melting temperature, composition of the alloy, energy stored at the time of cold plastic deformation, and the annealing time. The recrystallization temperature, most often, is defined as 0.35–0.6 of the absolute annealing temperature. For the analyzed alloys, assuming that the melting temperature for Cu-2Ni-1Si-0.8Ag alloy was approximately 1091 °C, the recrystallization temperature ranged from 210 to 550 °C.

Theoretically, the higher the draught coefficient, the lower the recrystallization temperature should be. For the analyzed Cu-2Ni-1Si and Cu-2Ni-1Si-0.8Ag alloys, the application of aging at a temperature of 500 °C (within 1 h) was closer to the upper limit of the recrystallization temperature range and did not allow for the elimination of the draught effects and restoration of the initial structure. This effect could be caused by the alloying additions or by contamination and impurities due to foreign atoms, which can be inhibiting. For the analyzed alloys, the presence of a Ni_2_Si phase was detected, which can be seen to have a different space orientation in Figure 7.

For the Cu-2Ni-1Si alloys with Ag addition, despite a lowering of the supersaturation temperature from 900 to 770 °C, which is below the temperature of the Cu + Ag eutectics, an increase in the properties took place. The supersaturation temperature for the alloys with Ag addition was lowered as the temperature above the eutectics caused Ag diffusion to the phase boundaries and a decrease in the material consistency due to plastic deformation. This is not the result of slipping and twinning but of deformation at the grain boundaries. This is a result of Ag segregation to the grain boundaries and stresses in the material built by positive forces, which are created by fixing the samples in the anvils of the Gleeble 3800 simulator.

The hardness and conductivity of the Cu-2Ni-1Si alloy is similar to the literature data [1,6,12,13,14,15]. As a result of the precipitated Cu-2Ni-1Si-0.8Ag alloy, the strengthening phase’s hardness after treatment amounted to 193HV. After heat treatment and cold plastic deformation, the hardness in the Cu-2Ni-1Si alloy increased by 60%, whilst the hardness in the alloy with Ag addition increased by 47%. The hardness increased as a result of the strengthening of the α phase in the matrix grains. After heat treatment and cold plastic deformation, the conductivity in the Cu-2Ni-1Si alloy increased by ~42%, whilst in the alloy with Ag addition it increased by 47%. The baseline conductivity of Cu-1Ni-1Si-0.8Ag alloy was slightly higher than that of the Cu-2Ni-1Si alloy. The changes in the hardness and conductivity are shown in Table 8, where it can be observed that the alloy with the addition of Ag after heat treatment and plastic deformation has a higher hardness by ~43 HV1 and higher conductivity by ~4 MS/m.

## 4. Conclusions

Our research on the effect of 0.8 mass% Ag addition to Cu-Ni-Si alloys has allowed us to draw the following conclusions:

1. Addition of 0.8 mass% Ag does not result in changes in the derivative curve of Cu2-Ni1-Si alloy, which may provide proof of dissolution of silver in the matrix at the time of crystallization.
2. After heat and plastic treatment, silver precipitates as small precipitates of about 20–180 nm, which results in the increase of hardening of the alloy compared to Cu-2Ni-1Si alloy.
3. Addition of the silver element does not cause changes in temperatures T_SOL_ and T_L_ in the Cu-2Ni-1Si alloy. Part of Ag during the crystallization process of the alloy dissolves in copper in the interdendritic spaces of the α matrix.
4. Conductivity of Cu-2Ni-1Si alloy after heat and plastic treatment is about 14 MS/m, while in the alloy with the addition of 0.8 mass% Ag, it increases to 17 MS/m so goes up 18%. Hardness in Cu-2Ni-1Si alloy after heat treatment and cold plastic treatment is about 150 HV; hardness of the alloy with Ag addition and after heat treatment and cold plastic treatment increases to 193 HV so goes up 22%.
5. The addition of silver at 0.8 mass% lowers the supersaturation temperature by 130 °C.

## Figures and Tables

**Table 1:** Chemical composition of the investigated copper alloys.

Sample Description	Elements as Compounds of the Investigated Cu Casts, Mass%			
	Ni	Si	Ag	Cu
Cu-2Ni-1Si	2	1	-	rest
Cu-2Ni-1Si-0.8Ag	2	1	0.8	rest

**Table 2:** The EDS spectrum analysis for the areas shown in Figure 4.

Element	Point #1 (wt.%)	Point #1 (at.%)	Point #2	Point #2 (at.%)
**Si**	-	-	17.1	30.7
**Ni**	-	-	54.9	47.1
**Cu**	100.0	100	28.0	22.2

**Table 3:** Results from thermal-derivative analyses of Cu-2Ni-1Si and Cu-2Ni-1Si-0.8Ag alloys.

Analyzed Alloy	Temperature, °C		Sample Mass, g
	T_L_	T_SOL_	
Cu-2Ni-1Si	1098	1057	11.36
Cu-2Ni-1Si-0.8Ag	1091	1060	11.33

**Table 4:** Latent heat of crystallization produced by phases and its percentage in the total heat of crystallization of Cu-2Ni-1Si alloy.

Cu-2Ni-1Si			
Heat Capacity in Liquid State Cp_l_, J/g °C	Heat Capacity in Solid State Cp_s_, J/g °C		Weight of Sample, g
0.448	0.392		11.36
Reaction	Latent Heat of Crystallization		Percentage, %
	Samples J	Unit Weight of a Sample, J/g	
L → α	1295.31	114.02	98.01
L → + Ni + Si	22.35	1.97	1.99
Total	1317.66	115.99	100

**Table 5:** Latent heat of crystallization produced by phases and its percentage in the total heat of crystallization of Cu-2Ni-1Si-0.6Ag alloy.

**Cu-2Ni-1Si-0.6Ag**			
**Heat Capacity in Liquid State Cp_l_,** **J/g °C**	**Heat Capacity in Solid State Cp_s_,** **J/g °C**		**Weight of Sample,** **g**
0.460	0.383		11.33
**Reaction**	**Latent Heat of Crystallization**		**Percentage,** **%**
	**Samples** **J**	**Unit Weight of a Sample,** **J/g**	
L → α+Ni+Si+Ag	1259.80	111.20	100

**Table 6:** Results of the EDS spectrum analysis for the areas from Figure 6.

Element	Area #1 (Figure 6a) (wt.%)	Area #1 (Figure 6a) (at.%)	Point #1 (Figure 6c) (wt.%)	Point #1 (Figure 6c) (at.%)	Point #2 (Figure 6c) (at.%)	Point #2 (Figure 6c) (wt.%)
Si	1.2	2.7	1.5	04.6	12.7	25.0
Ni	2.7	2.8	5.2	07.5	37.1	34.9
Ag	1.2	0.7	66.1	51.8	9.4	4.8
Cu	94.9	93.8	27.2	36.1	40.7	35.3

**Table 7:** Results of the EDS spectrum analysis for the points from Figure 7b.

Element	Point #1 (wt.%)	Point #1 (at.%)	Point #2 (wt.%)	Point #2 (at.%)	Point #3 (wt.%)	Point #3 (at.%)	Point #4 (wt.%)	Point #4 (at.%)
Si	2.3	5.1	1.5	3.3	1.3	2.9	6.2	12.9
Ni	7.1	7.4	5.1	5.4	4.9	5.3	23.4	23.2
Ag	2.9	1.7	2.7	1.6	1.6	0.9	1.8	1.0
Cu	87.7	85.8	90.7	90.7	92.1	90.8	68.6	62.9

**Table 8:** Averaged results obtained from the hardness measurements using the Vickers method and electrical conductivity (HT+CPD: after heat treatment plus cold plastic deformation).

The Samples	Conductivity MS/m	Standard Deviation	Average Hardness, HV1	Standard Deviation
Cu-Ni-Si	8	0.01	60	1.68
Cu-Ni-Si HT+CPD	14	0.06	150	6.02
Cu-Ni-Si-Ag	9	0.25	90	7.44
Cu-Ni-Si-Ag HT+CPD	17	0.15	193	7.03
